# Bidirectional associations between workplace bullying and sickness absence due to common mental disorders – a propensity-score matched cohort study

**DOI:** 10.1186/s12889-024-18214-5

**Published:** 2024-03-08

**Authors:** Rebecka Holmgren, Alessandra Grotta, Kristin Farrants, Linda L. Magnusson Hanson

**Affiliations:** 1 Stress Research Institute, Division of Psychobiology and Epidemiology, Department of Psychology, Stockholm University, Stockholm, Sweden; 2 Department of Public Health Sciences, Stockholm University, Sweden & Centre for Health Equity Studies, Stockholm University/Karolinska Institutet, Stockholm, Sweden; 3 Division of Insurance Medicine, Department of Clinical Neuroscience, Karolinska Institutet, Stockholm, Sweden

**Keywords:** Bullying, Sick leave, Mental disorders, Occupational stress, Propensity score

## Abstract

**Background:**

The link between workplace bullying and poor mental health is well-known. However, little is known about the prospective and potentially reciprocal association between workplace bullying and mental health-related sickness absence. This 2-year prospective study examined bidirectional associations between exposure to workplace bullying and sickness absence due to common mental disorders (SA-CMD) while controlling for confounding factors from both work and private life.

**Methods:**

The study was based on propensity score-matched samples (*N* = 3216 and *N* = 552) from the Swedish Longitudinal Occupational Survey of Health, using surveys from years 2012, 2014 and 2016. Self-reported exposure to workplace bullying was linked to registry-based information regarding medically certified SA-CMD (≥ 14 consecutive days). The associations were examined by means of Cox proportional hazards regression and via conditional logistic regression analysis. Hazard ratios and odds ratios with 95% confidence intervals were estimated.

**Results:**

Exposure to workplace bullying was associated with an increased risk of incident SA-CMD (HR: 1.3, 95% CI: 1.0–1.8), after accounting for the influence of job demands, decision authority, previous SA-CMD, as well as other sociodemographic covariates. However, we found no statistically significant association between SA-CMD and subsequent workplace bullying (OR 1.2, 95% CI 0.7–1.9).

**Conclusions:**

The results support an association between self-reported workplace bullying and SA-CMD, independent of other sociodemographic factors and workplace stressors. Preventing workplace bullying could alleviate a share of the individual and societal burden caused by SA globally.

**Supplementary Information:**

The online version contains supplementary material available at 10.1186/s12889-024-18214-5.

## Introduction

Mental disorders are a major cause of sickness absence globally [1, 2] and impose a substantial economic burden for society, partly through associated benefit payments [3]. In order to reduce these societal costs as well as increase the occupational ability of working-aged individuals, it is of utmost importance to understand risk factors contributing to sickness absence due to mental disorders.

Workplace bullying has a global prevalence rate of 10–15% within the working population [4]. It is defined as being repeatedly exposed to negative social behaviours at work over a prolonged period of time, to which one feels unable to defend oneself [5]. Workplace bullying has consistently been associated with adverse mental health consequences, such as symptoms of stress, anxiety, and depression [6–9]. Furthermore, workplace bullying is associated with impairment in physical health [10, 11] and associated with all cause-sickness absence [12–16]. Reverse relationships have also been demonstrated, with both poor mental health and all-cause sickness absence increasing the risk of exposure to bullying, thus indicating a potential vicious circle [13, 17].

Conceptually, sickness absence after exposure to bullying can be understood either as a means of coping with an adverse working situation [18] or as a consequence resulting from deteriorated health [12, 14, 16]. Perceived stress [19], poor sleep [20] and general mental distress [21] have been found to mediate the association between workplace bullying and sickness absence. These symptoms are part of the diagnostic criteria for several common mental disorders, making it plausible to also assume an association between workplace bullying and subsequent sickness absence specifically due to mental disorders (SA-CMD). Accordingly, a Norwegian study recently found that one out of three individuals reporting struggles with work participation due to common mental disorders had experienced workplace bullying [18].

However, studies on the prospective association between exposure to workplace bullying and SA-CMD among representative working populations are scarce and no attention has been given to the possible reverse association. Results from the two studies known to us indicate that workplace bullying is a risk factor for SA-CMD within one year following exposure [22, 23]. However, mental health disorders following workplace bullying might take time to develop and manifest [14, 16], indicating the need for studies applying longer follow-up periods in order to capture its effect on work ability. Additionally, the amount of demands and available resources at work may influence both the risk of becoming exposed to workplace bullying and the risk of SA-CMD [24, 25] and thus may need to be accounted for.

The objective of this study is therefore to examine the 2-year prospective association between exposure to workplace bullying and SA-CMD, and between SA-CMD and workplace bullying, taking potential confounding from both sociodemographic and work factors into consideration. In addition, the potential dose–response relationship between frequency of exposure and SA-CMD will be explored.

## Methods

### Data sources

The study sample was drawn from participants in the Swedish Longitudinal Occupational Survey of Health (SLOSH), a longitudinal study focusing on associations between work and health [26]. Data stem from SLOSH surveys and national registers, linked via personal identification numbers. Information on sickness absence and disability pension was obtained from the Swedish Social Insurance Agency’s Microdata for the Analysis of Social Insurance register. Information on date of death was retrieved from the Cause of Death Register. Information on retirement was retrieved from Statistics Sweden.

SLOSH was initiated in 2006 with follow-up questionnaires sent out biennially, thus far comprising 57 105 individuals. The initial SLOSH follow-up was directed to respondents (response rate 65.4%) from the Swedish Working Environment Study (SWES) 2003. Since then, the SLOSH cohort has expanded with additional samples of respondents to SWES 2005–2019. SWES in turn consists of gainfully employed individuals of age 16 to 64, sampled from the total population in Sweden using stratified random selection [26].

**Fig. 1:**
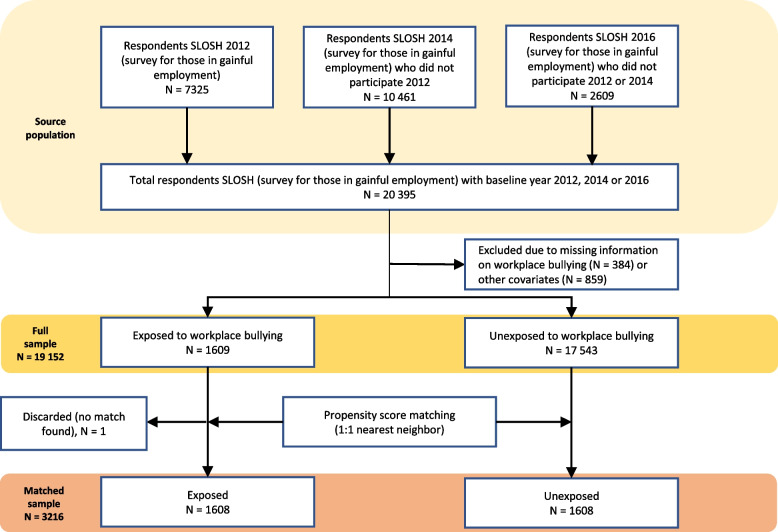
Flow chart of the sample selection process. SLOSH = Swedish Longitudinal Occupational Survey of Health

**Fig. 2:**
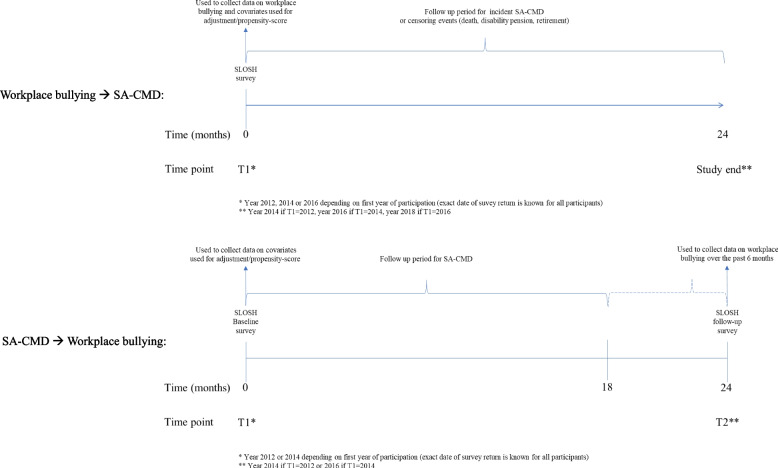
**a** Study design in order to examine the association between exposure to workplace bullying and subsequent SA-CMD. **b** Study design in order to examine the association between SA-CMD and subsequent workplace bullying

Until 2020, all SLOSH-participants received two versions of the questionnaire, one directed towards those working 30% or more of full-time during the past 3 months and one for those not currently working/working less than 30% of full-time. For our main analysis, “in-work” questionnaires from 2012, 2014 and 2016 were pooled and used (*N* = 20,395, response rate 50.9–56.7% each year). In cases of repeated participation, the first year of participation was used. We restricted the sample to individuals with available information on all covariates. The excluded cases (with partial missingness) were to a slightly higher degree men, born outside of Sweden, holding temporary positions and without SA-CMD during follow-up. The proportion of partial missingness did not differ by bullying status. This resulted in a study sample of 19,152 individuals. A flow chart of the selection process is presented in Fig. 1 and the study design is presented in Fig. 2a.

In order to examine the association between SA-CMD and workplace bullying, we reorganized the data set, creating a sample (*N* = 10 932) consisting of all individuals who answered two consecutive in-work questionnaires (2012 and 2014 or 2014 and 2016, see study design in Fig. 2b and flow chart of selection process in Supplementary Figure 1 in Additional file 1).

### Jurisdictional context

Residents in Sweden, aged 16 or older and having income from work or unemployment benefits, are entitled to sickness absence benefits from the Social Insurance Agency if unable to work due to disease or injury. A medical certificate including a primary diagnosis (according to the International Classification of Diseases [ICD]), issued at the latest at day 7 of the sickness spell, must be provided. Benefits are usually given from day 15 of the sickness spell, with the employer covering the first 13 days after an unpaid waiting day.

### Sickness absence due to common mental disorders

We obtained information on start date and medical cause of sickness absence spells during the period November 1, 2002 to November 29, 2018. We defined SA-CMD as having at least one spell of sickness absence (> 14 consecutive days), with the primary diagnosis being mood/affective disorders (ICD-10: F.30–39) or neurotic, stress-related, and somatoform disorders (ICD-10: F40-F48). Time (measured in months) from survey return to incident spell of SA-CMD during a 2-year follow-up period was used as outcome variable in our main analysis. In the SA-CMD to workplace bullying analysis, the occurrence of at least one incident spell of SA-CMD (here used as exposure variable) was measured using a follow-up period of 18 months, starting from the time of first survey return and ending 6 months before the second survey return (as to not overlap with the timing of the outcome).

### Workplace bullying

Exposure to workplace bullying was assessed in SLOSH through the self-labelling method [5] using the following question: *“During the last 6 months, have you been subjected to personal persecution in the form of unkind words or behaviors from superiors or fellow workers?”* Response alternatives were *“Yes, one or several times a week”*, *“Yes, one or several times a month”*, *“Yes, sometime during the last 6 months”* and *“No”*. A binary variable, with affirmative responses indicating exposure, was used for the main analyses. For the dose–response analyses, response categories were grouped into no/occasional/frequent exposure (considered frequent if indicating weekly or monthly exposure).

### Statistical analysis

For the association between workplace bullying and incident SA-CMD, the selection of confounders was guided by a directed acyclic graph (DAG, see Supplementary Figure 2 in Additional file 1) which was drawn based on prior research. In order to reduce health-related selection bias and address confounding, we applied propensity score matching to account for confounders [27, 28]. With this approach, the hazard rate (HR) represents the risk of SA-CMD after exposure to workplace bullying for employees who were actually bullied at work (i.e. the “average treatment effect for the treated” [29]). The propensity score was generated using a multivariate logistic regression model. Several specifications of the logistic model were tested and evaluated based on their balancing properties [30]. In the final propensity score model, we adjusted for the minimum set of confounders needed to estimate the association between workplace bullying and SA-CMD and additionally included age and covariates only related to the outcome, as well as interaction terms between age and all other included covariates. This resulted in a propensity score using 5 registry-based covariates: sex (male/female); age (≤ 35/36–45/46–55/56–65/ > 65 years); country of birth (Sweden/outside Sweden); socioeconomic position (6 groups) and SA-CMD prior to survey return (using information since 2002), and 6 covariates from SLOSH: married or cohabiting (yes/no); cohabiting with children (yes/no); job demands and decision authority score (measured using the mean of 4 and 3 items from the Job-Demand-Questionnaire respectively); contract type (permanent/temporary) and a variable indicating participant’s baseline year (2012, 2014, 2016) in order to account for potential contextual effects affecting the likelihood of receiving sickness absence benefits. Covariates were measured at baseline. Based on the estimated propensity score, exposed individuals were matched to non-exposed individuals, using 1:1 nearest neighbor matching without replacement, within the recommended caliper width of 0.2 of the standard deviation of the logit of the propensity score [31]. We checked the balancing properties of the propensity score by comparing standardized differences between exposed and non-exposed individuals in the matched and unmatched sample (using a threshold of < 10% to indicate good balance [30]). Statistical tests (chi-square tests and t-tests) comparing differences in covariates between exposed and non-exposed were performed for completeness only, but not used for balance evaluation since they can be affected by sample size. We then fitted Cox proportional hazards regression models on the propensity score-matched sample using months since survey return as time-scale. Employees were followed for 2 years (24 months) until incident SA-CMD, with censoring in case of study end, death, receiving full-time disability pensioning or at retirement (using data on main income source), whatever occurred first. It should be noted that information on retirement was only available until end of November 2016. We checked the assumption of proportional hazards by visually inspecting log–log survival plots and by using Schoenfeld’s global test. We present crude hazard ratios (HR) and their 95% confidence intervals (CI). To account for the matched nature, the variance was clustered by matched sets [29]. We examined dose–response relationship by comparing level-specific HRs retrieved from Cox models. A *p*-value for trend was obtained through linear modelling of the categorical exposure. Sensitivity analysis involved excluding individuals with prior SA-CMD from the sample before carrying out the propensity score matching, in order to address confounding from mental health disorders.

For the association between SA-CMD and subsequent workplace bullying, we again selected confounders using a DAG (see Supplementary Figure 3 in Additional file 1) based on prior research and generated a propensity score using a multivariate logistic regression model. Covariates used in the final propensity score model for this analysis are presented in Supplementary Table 1 in Additional file 1. Among other factors, we adjusted for baseline bullying status. Additionally, this propensity score included interaction terms between age and all other covariates. We matched exposed and non-exposed individuals based on their propensity score, using the same matching procedure as explained above. We then fitted conditional logistic regression models on the propensity score-matched sample. Results are presented as odds ratios (OR), together with their 95% CI, again clustering the variance by matched sets. A sensitivity analysis involved excluding individuals exposed to workplace bullying at baseline from the sample before carrying out the propensity score matching in order to address confounding from baseline bullying status.

For both associations, we performed additional analyses in the full samples, addressing confounders through regression adjustment. We adjusted for the same confounders that were included in the propensity scores. Thus, we implemented a Cox proportional hazards regression model to investigate the association between workplace bullying and incident SA-CMD while adjusting for the selected confounders (here, previous SA-CMD was adjusted for through stratification in order to not violate the proportional hazards assumption) and a multivariate logistic regression model to assess the association between SA-CMD and subsequent workplace bullying, while adjusting for confounders.

All statistical analyses were carried out in STATA (version 16), using psmatch2 command for the matching procedure [32]. Two-sided *p*-values lower than 0.05 were considered statistically significant.

### Ethical considerations

Ethical approval was obtained for SLOSH from the Regional Ethical Review Board in Stockholm [#2012/373‐31/5, #2006/158‐31, #2008/240‐32, #2008/1808‐32, #2010/0145‐32, #2012/373‐31/5, #2013/2173‐32, #20152187, #2015/2298‐32, #2017/25‐35‐32]. All participants received written information about the purpose of SLOSH and informed consent has been obtained for each survey year.

## Results

### Baseline characteristics

Demographic characteristics of exposed and unexposed individuals in full and propensity score (PS)-matched samples are presented in Table 1. A balanced distribution of all baseline covariates was reached in the PS-matched sample. Distribution of baseline covariates by frequency of exposure is provided in Supplementary Table 2a-b in Additional file 1.

**Table 1:** Baseline characteristics of exposed and unexposed subjects in full sample and propensity score matched sample

Variable	Full sample (*N* = 19,152)			*P*-value	Propensity score matched sample (*N* = 3216)			*P*-value
	Exposed (*N* = 1609) %(N)	Unexposed (*N* = 17,543) %(N)	Standardized difference (%)		Exposed (*N* = 1608) %(N)	Unexposed (*N* = 1608) %(N)	Standardized Difference (%)	
**Female**	65.2(1049)	55.5(9739)	19.9	< 0.001	65.2(1048)	67.4(1083)	4.6	0.19
**Age group**			10.1	0.01			3.9	0.88
≤ 35	7.6(122)	8.7(1530)			7.6(122)	6.9(111)		
36–45	21.5(346)	21.4(3747)			21.5(345)	21.6(348)		
46–55	33.4(538)	31.5(5522)			33.5(538)	34.2(550)		
56–65	34.4(553)	33.6(5900)			34.4(553)	34.6(556)		
> 65	3.1(50)	4.8(844)			3.1(50)	2.7(43)		
**Born in Sweden**	89.8(1445)	93.8(16,449)	14.4	< 0.001	89.8(1444)	91.0(1463)	4.0	0.26
**Married/cohabiting**	70.8(1139)	80.3(14,095)	22.4	< 0.001	70.8(1139)	72.1(1160)	2.9	0.41
**Cohabiting with children**	47.2(760)	48.0(8426)	1.6	0.54	47.3(760)	48.0(772)	1.5	0.67
**Socioeconomic position**			14.0	< 0.001			2.1	0.99
Unskilled worker	18.5(297)	14.4(2521)			18.4(296)	18.8(303)		
Skilled worker	17.2(277)	15.9(2795)			17.2(277)	16.7(269)		
Assistant non-manual employee	14.0(225)	13.5(2360)			14.0(225)	13.6(218)		
Intermediate non-manual employee	30.8(496)	32.9(5772)			30.9(496)	31.2(501)		
Professional/upper level executive and self-employed^a^	19.5(314)	23.3(4095)			19.5(314)	19.7(317)		
**Permanent position**	98.6(1587)	97.8(17,162)	6.1	0.03	98.6(1586)	98.6(1586)	< 0.1	0.99
**Job demands, mean (SD) (range 1–5)**	2.9(0.5)	2.6(0.6)	54.1	< 0.001	2.9(0.5)	2.9(0.5)	1.2	0.74
**Decision authorithy, mean (SD) (range 1–5)**	2.8(0.8)	3.1(0.7)	34.2	< 0.001	2.9(0.8)	2.8(0.8)	1.5	0.66
**LTSA-CMD prior to baseline**	20.6(332)	11.0(1936)	26.5	< 0.001	20.6(331)	19.5(314)	2.6	0.45
**Baseline year**			1.3	0.88			3.3	0.64
2012	36.7(591)	36.1(6338)			36.7(590)	37.9(609)		
2014	50.8(818)	51.2(8983)			50.9(818)	50.6(814)		
2016	12.4(200)	12.7(2222)			12.4(200)	11.5(185)		

^a^Grouped together due to low N of subjects being self-employed

In the full sample, 8.4 percent (*N* = 1609) were exposed to workplace bullying, and all but one of these were included in the matched sample. Mean follow-up time was 23.0 months (SD: 3.7) and 23.2 months (SD: 3.3) for the PS-matched and full sample respectively. See Fig. 4a-b in Additional file 1 for of distribution of events in time.

### Workplace bullying and incident SA-CMD

We found a statistically significant association between exposure to workplace bullying and incident SA-CMD during a follow-up period of 2 years. In our PS-matched sample, individuals exposed to workplace bullying had a 30% greater hazard of SA-CMD during follow-up (HR: 1.3, 95% CI: 1.0–1.8, *p* = 0.03). A significant linear trend between frequency of exposure to bullying and SA-CMD was also observed (p^trend^ < 0.01). When comparing level-specific HRs, individuals frequently exposed to workplace bullying had the greatest hazard of SA-CMD. The results are presented in Table 2. Excluding individuals with prior SA-CMD (*N* = 2268) before matching resulted in similar results regarding the risk of SA-CMD after exposure to workplace bullying (HR: 1.4, 95% CI: 0.9–2.0, *p* = 0.10).

**Table 2:** Hazard rates (HR) with 95% confidence intervals (CI) from Cox proportional hazards models of the association between exposure to workplace bullying and incident sickness absence due to common mental disorders (SA-CMD), using the propensity score-matched sample^ab^

	N	Cases SA-CMD	HR (95% CI)	*P*-value
Occurrence of workplace bullying				
*No*	1608	87	Ref	
*Yes*	1608	116	1.3 (1.0–1.8)	0.03
Frequency of exposure to workplace bullying				
*Never*	1608	87	Ref	
*Occasional*	1251	83	1.2^c^ (0.9–1.7)	0.16
*Frequent*	357	33	1.7^c^ (1.1–2.5)	< 0.01

^a^Matched on the following covariates: sex, age, birth country, marital status, cohabiting with children, socioeconomic position, contract type, job demands, decision authority, prior SA-CMD and baseline year

^b^Variance clustered by matched sets to account for matching

^c^p_trend_ < 0.01

Models performed on the full sample also showed similar results (adjusted HR: 1.4 95% CI 1.1–1.7, *p* < 0.01). The dose–response analysis revealed a clear gradient with increasing HR of SA-CMD as the frequency of bullying increased (p_trend_ < 0.01, see Supplementary Table 3 in Additional file 1).

### SA-CMD and workplace bullying

In the full sample, 2.5% (*N* = 276) were exposed to at least one episode of SA-CMD between T1 and T2 (either 2012–2014 or 2014–2016), and were matched to an unexposed individual. Characteristics of the samples are presented in Supplementary Table 1 in Additional file 1. No statistically significant association between SA-CMD and later exposure to workplace bullying was found when accounting for potential confounding variables (PS-matched sample *N* = 552, OR 1.2, 95% CI 0.7–1.9, *p* = 0.53; full sample *N* = 10,932, adjusted OR 1.4, 95% CI 0.9–2.0, *p* = 0.10). When restricting the sample to individuals who had not been exposed to bullying at T1, we obtained similar results (OR 1.0, 95% CI 0.5–1.7, *p* = 0.88).

## Discussion

In this large cohort study, we found an association between exposure to workplace bullying and incident sickness absence due to common mental disorders using a 2-year follow-up period. The association was robust to multiple statistical approaches and remained after accounting for confounding from several sociodemographic and work-related factors. We found no strong support for an association between sickness absence due to common mental disorders and subsequent workplace bullying.

Our findings are consistent with those of Janssens et al. [23] and Stromholm et al. [22] who also found an increased risk of SA-CMD after exposure to workplace bullying, using a 1-year follow-up period. Contrary to their studies, we were able to control for the influence from other work-related factors such as job demands and decision authority. The fact that the association remains after such control suggests that exposure to workplace bullying might negatively influence one’s work ability.

Interestingly, in our study, the association between exposure to workplace bullying and SA-CMD was similar over a 2-year follow-up period. This long risk period might be explained by the persistent and escalating bullying process itself [5], eventually leading up to sickness absence as one way of coping with the situation for the exposed. The long risk period might further be explained by the fact that health consequences such as common mental disorders may take time to develop and not necessarily hinder one’s work ability immediately. The process from becoming ill at work to go on sick leave (and eventually return to work) has been described as a staged process [33], where each step also requires overcoming barriers such as to recognize symptoms and to seek professional help. In addition, work demands, as well as workplace policies and actions might affect the route from exposure to workplace bullying to taking leave of absence [34]. Our result thus supports the understanding of sickness absence as a function of the interplay of self-rated health and help-seeking behavior, objective health status as well as available coping resources at work [33, 35], and not only as a mere manifestation of poor mental health. We encourage future studies to more closely examine this pathway, in order to establish mediating factors.

Regarding the strength of the association, our results indicate a small effect size of workplace bullying on SA-CMD [36]. The small effect size is on par with effect sizes of other psychosocial stressors at work on mental health-related sickness absence [24]. Even though the risk estimate itself might be considered modest, recent research has pointed to the fact that cumulative exposure to several psychosocial job stressors of this magnitude might dramatically increase the risk of SA [37], underlining the importance of preventing any of these job stressors. The results from our dose–response analyses indicate that a more frequent exposure to workplace bullying might be more strongly related to subsequent SA-CMD, thus pointing at a potential vulnerable group. This result needs however be interpreted with caution, as the group reporting frequent exposure was small.

Despite the fact that a reverse link has been observed between all-cause SA and workplace bullying [13, 38], as well as between mental health symptoms and workplace bullying [17], we did not find any support for SA-CMD being a risk for later exposure to workplace bullying. One interpretation of this is that the affected employees improve their mental health during their period of sickness absence, and therefore the increased risk of subsequent bullying diminishes. Another, not mutually exclusive, interpretation is that the link between all-cause SA and workplace bullying is mainly driven by SA due to other reasons than common mental disorders. Our results thus indicate that the relationship between workplace bullying and SA-CMD is unidirectional. As far as we know, our study is the first to examine this relationship and more research is needed to rule out the possibility of reciprocity.

### Study strengths and limitations

This prospective study is the first to extensively account for the influence of job stressors (in addition to other sociodemographic variables) on the relationship between workplace bullying and SA-CMD. We do so by using propensity-score matching to create a sample that is more comparable (in terms of reduced observed confounding) than comparing exposed and unexposed in the original full sample.

Our results need to be interpreted in light of the study limitations. Regarding our analytical approach, it is important to point out that the estimated propensity score relies on observable data only, and therefore residual confounding from unmeasured covariates (which in the bullying-SA-CMD-relationship could be caused by factors such as personality traits or childhood experiences) can still exist, which could have caused us to overestimate the associations. A drawback of using propensity-score matching is the reduction in sample size, resulting in increased variance. With these limitations in mind, the fact that our findings are consistent across multiple statistical methods suggests that there is a link between workplace bullying and SA-CMD. More studies, applying a causal approach, are needed in order to confirm this link. Preferably, such studies could also include repeated measures of the exposure.

The use of self-reported data comes with a risk of information bias [39]. Here, misclassification of exposure might have affected our estimates, although our sensitivity analysis excluding individuals with prior SA-CMD (who might have a biased perception of their social surrounding/work environment) to some extent should reduce this risk. The use of a registry-based outcome variable further reduces the risk of common method bias. Although the use of a registry-based outcome variable ensured that no loss to follow-up occurred, our initial sample might still be affected by selection bias, since prior research has shown that women and individuals who are married, older, or have higher educational attainment are overrepresented in the SLOSH-cohort [26]. Additionally, using full cases only might have introduced selection bias. This somewhat restricts the generalizability of our findings to the whole working population in Sweden.

Given that the measure of workplace bullying reflects a period of 6 months prior to the survey, we might have missed cases of SA-CMD that appear close in time to exposure and ended before survey return. Additionally, we did not consider ongoing part-time sickness absence spells at the time of survey return. This could have led to an underestimation of the strength of the association. As discussed above, the process from exposure to sick leave is likely to take time, and therefore we do not expect such potential misclassification to have a significant impact on our findings.

We chose to restrict our main analyses to incident SA-CMD only and to not distinguish between full- or part-time sickness absence. Our measure of SA-CMD does not encompass information regarding the duration of SA-CMD, which could have further nuanced our findings. Additionally, we did not censor for competing events, such as sickness absence due to other reasons than common mental disorders. Given that workplace bullying is an established risk factor for physical illness [10, 11], individuals in our sample might have been wrongly categorized into being at risk of SA-CMD, when in fact, they were not at risk since they were already on sick leave due to other causes. If so, the associations we have found might be underestimated.

We did not have information regarding whether individuals in our sample changed job, which could have affected their mental health status and, by extension, their likelihood of SA-CMD. A recent Swedish study found that individuals who had been exposed to workplace bullying were more likely to change jobs. Their findings further suggested that while changing job might reduce levels of anxiety among those exposed to bullying, it did not significantly alter their levels of depressive symptoms [40]. It is thus possible that the use of other coping strategies (such as changing job) might influence the bullying-SA-CMD association.

Lastly, legal regulations regarding sickness absence differ across time periods and countries, with Sweden being among the more generous countries with regards to sickness absence benefits [41]. Although we adjusted for baseline year in order to account for any national time trends with regards to sickness absence, our results might not be generalizable to employees in countries that apply very different sickness absence policies.

## Conclusions

Our results support a prospective association between self-reported workplace bullying and sickness absence due to common mental disorders, also when accounting for sociodemographic factors and workplace stressors. Organizations should be informed about the risk of hampered work ability among employees who have been exposed to workplace bullying. Continued preventive measures against workplace bullying are recommended as they may alleviate a share of the individual and societal burden caused by sickness absence.

### Supplementary Information

**Additional file 1.** Supplementary material.

## Data Availability

The datasets generated and analyzed during the current study are not publicly available due to ethical restrictions and considering that sensitive personal data are involved. Access to the data may be provided to other researchers in line with Swedish law and after consultation with the Stockholm University legal department. Requests for data, stored at the Stress Research Institute, Department of Psychology, Stockholm University should be sent to data@slosh.se.

## Abbreviations

SA-CMD: Sickness absence due to common mental disorders
SLOSH: The Swedish Longitudinal Occupational Survey of Health
SWES: Swedish Working Environment Study
ICD: International Classification of Diseases
DAG: Directed Acyclic Graph
HR: Hazard rate
CI: Confidence interval
OR: Odds ratio
PS: Propensity score
