# Supplementary material for: Bidirectional associations between workplace bullying and sickness absence due to common mental disorders – a propensity-score matched cohort study
Source: BMC Public Health. 2024 Mar 8;24:744. doi: 10.1186/s12889-024-18214-5 (PMC10921817; doi:10.1186/s12889-024-18214-5)
Supplement: Supplementary file 1 — Additional file 1. Supplementary material. [file 12889_2024_18214_MOESM1_ESM.pdf]

Supplementary material

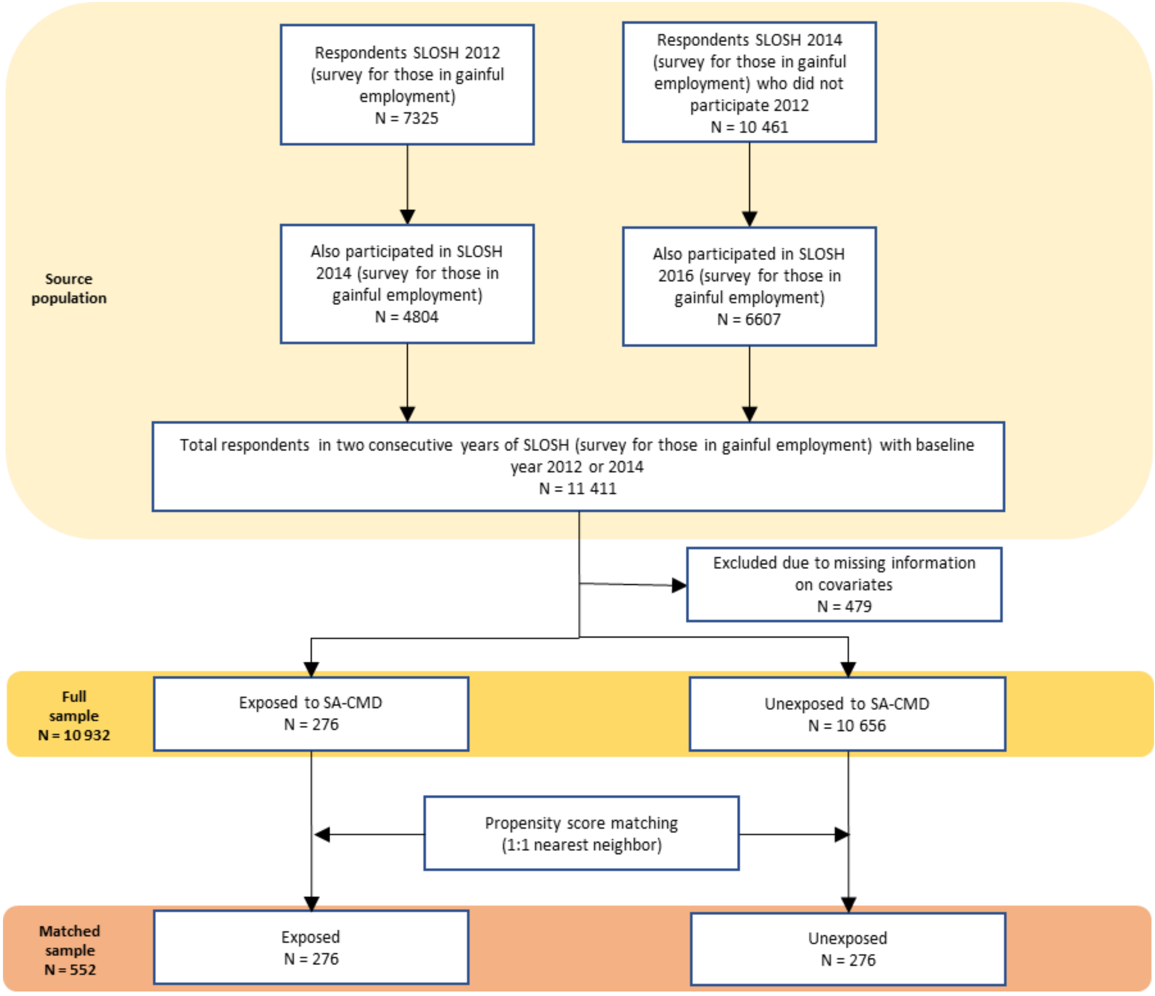

Supplementary figure 1. Flowchart of selection process for the sample used for analysis of the relationship between sickness absence due to common mental disorders (SA-CMD) and subsequent exposure to workplace bullying.

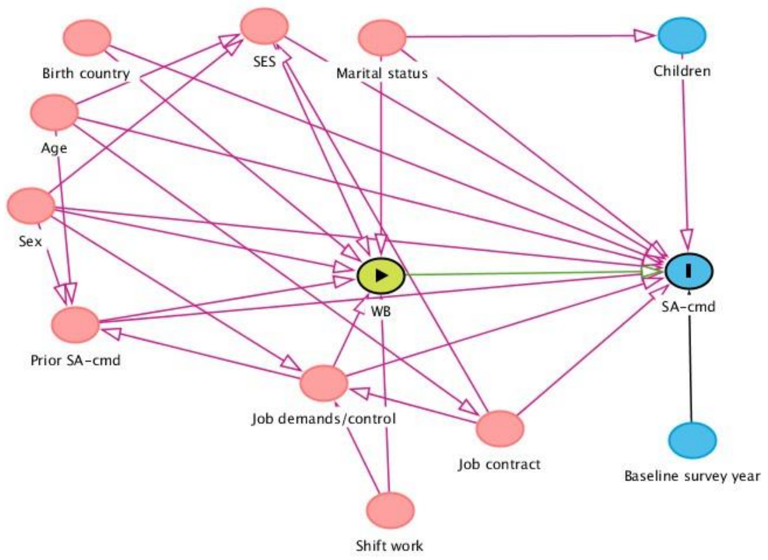

Supplementary figure 2. Directed acyclic graph displaying the postulated relationships between workplace bullying, sickness absence due to common mental disorders (SA-cmd) and relevant covariates, used to guide our logistic model for the propensity score. SA-cmd=Sickness absence due to common mental disorders, SES = socioeconomic status, WB=workplace bullying

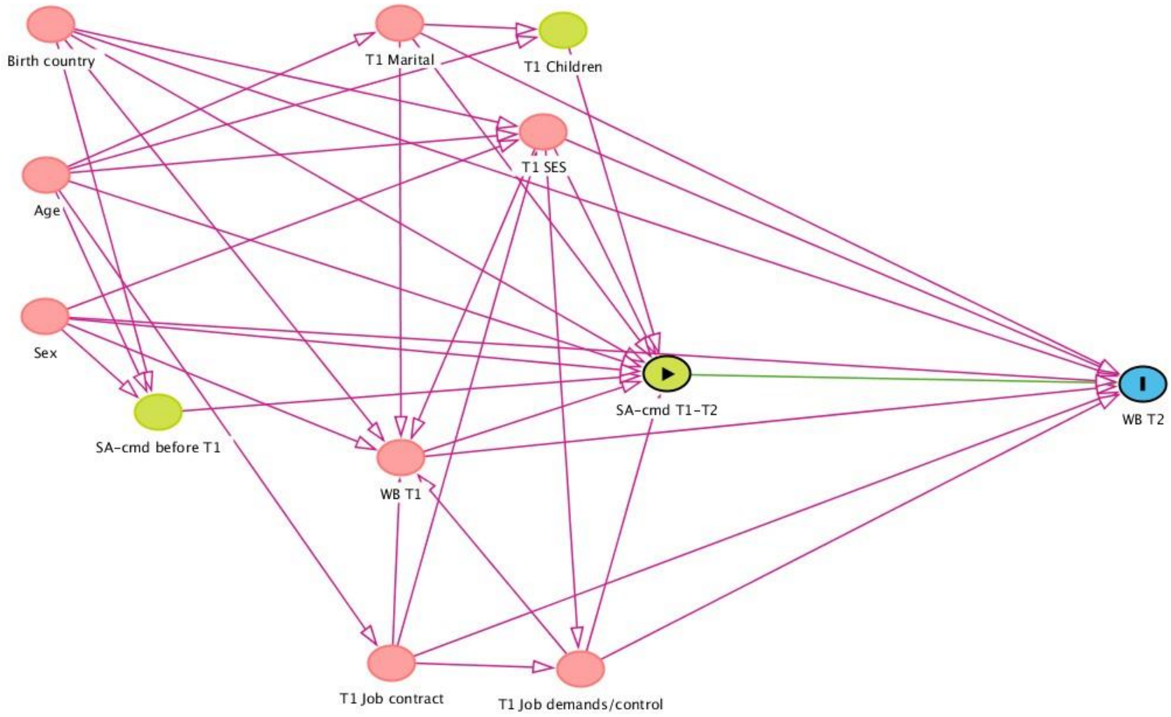

Supplementary figure 3. Directed acyclic graph displaying the postulated relationships between history of sickness absence due to common mental disorders (SA-cmd), workplace bullying (at baseline and follow-up) and relevant covariates (at baseline), used to guide our logistic model for the propensity score. SA-cmd=Sickness absence due to common mental disorders, SES = socioeconomic status, WB=workplace bullying

.

Supplementary table 1. Baseline characteristics of exposed and unexposed subjects in full sample and propensity score matched sample used for the analysis of reverse association. Presented as % (N) or mean (standard deviation).

	Full sample (N=10932)				Propensity score matched sample (N=552)			
Variable	Exposed (N=276)	Unexposed (N=10656)	Standardized difference (%)	p-value	Exposed (N=276)	Unexposed (N=276)	Standardized Difference (%)	p-value
**Female**	80.8 (223)	57.4 (6121)	52.2	<0.01	80.8 (223)	80.4 (222)	0.1	0.91
**Age group**			5.0	0.71			6.9	0.72
≤45	27.9 (77)	27.3 (2904)			29.7 (82)	29.7 (82)		
46-55	36.2 (100)	34.5 (3676)			36.2 (100)	33.0 (91)		
>55	35.9 (99)	38.3 (4076)			35.9 (99)	37.3 (103)		
**Born in Sweden**	90.9 (251)	93.7 (9984)	10.3	0.07	90.9 (251)	92.8 (256)	6.6	0.44
**Married/cohabiting**	76.1 (210)	79.6 (8582)	8.4	0.15	76.1 (210)	75.0 (207)	2.5	0.77
**Socioeconomic position**[a]			1.3	0.83			1.6	0.85
Unskilled worker	10.1 (28)	13.6 (1449)			10.1 (28)	13.4 (37)		
Skilled worker	19.6 (54)	15.5 (1654)			19.6 (54)	15.6 (43)		
Assistant non-manual employer	17.4 (48)	13.0 (1381)			17.4 (48)	12.0 (33)		
Intermediate non-manual employer	29.7 (82)	34.2 (3641)			29.7 (82)	35.1 (97)		
Professional/upper level executive and self-employed[b]	23.2 (64)	23.8 (2531)			23.2 (64)	23.9 (66)		
**Permanent position**	97.5 (269)	98.2 (10465)	5.1	0.36	97.5 (269)	96.7 (267)	4.3	0.61
**Job demands, mean (SD) (range 1-5)**	2.8 (0.6)	2.6 (0.5)	31.5	<0.01	2.8 (0.6)	2.8 (0.6)	1.1	0.90
**Decision authorithy, mean (SD) (range 1-5)**	3.0 (0.7)	3.1 (0.7)	19.8	<0.01	3.0 (0.7)	2.9 (0.7)	2.2	0.80
**Exposed to workplace bullying at T1**	13.8 (38)	8.0 (850)	18.7	<0.01	13.8 (38)	14.5 (40)	2.1	0.81

[a]Used as manual/non-manual worker when adjusting/including in propensity score and in tests of differences

[b]Grouped together due to low N of subjects being self-employed

Supplementary table 2a. Baseline characteristics by frequency of exposure using full study sample. Presented as % (N) or mean (standard deviation).

Variable	Unexposed (N=17,543)	Occasionally exposed (N=1252)	Frequently exposed (N=357)	p-value from tests of difference[a]
**Female**	55.5(9739)	65.3(818)	64.7(231)	<0.001
**Age group**				0.03
<35	8.7(1530)	7.6(95)	7.6(27)	
36-45	21.4(3747)	20.8(260)	24.1(86)	
46-55	31.5(5522)	33.2(416)	34.2(122)	
56-65	33.6(5900)	35.0(438)	32.3(115)	
>65	4.8(844)	3.4(43)	2.0(7)	
**Born in Sweden**	93.8(16449)	89.7(1123)	90.2(322)	<0.001
**Married/cohabiting**	80.3(14095)	70.8(886)	70.9(253)	<0.001
**Living with ≥1 child**	48.0(8426)	46.8(586)	48.7(174)	0.67
**Socioeconomic position**				<0.001
Unskilled worker	14.4(2521)	18.1(227)	19.6(70)	
Skilled worker	15.9(2795)	17.3(216)	17.1(61)	
Assistant non-manual employer	13.5(2360)	14.0(175)	14.0(50)	
Intermediate non-manual employer	32.9(5772)	31.0(388)	30.3(108)	
Professional/upper level executive and self-employed[b]	23.3(4095)	19.6(246)	19.0(68)	
**Permanent position**	97.8(17162)	98.7(1236)	98.3(351)	0.09
**Job demands (1-5)**	2.6(0.6)	2.8(0.5)	3.0(0.6)	<0.001
**Decision authorithy (1-5)**	3.1(0.7)	2.9(0.8)	2.8(0.8)	<0.001
**SA-cmd prior to baseline**	11.0(1936)	19.7(247)	23.8(85)	<0.001
**Baseline year**				0.51
2012	36.1(6338)	35.8(448)	40.1(143)	
2014	51.2(8983)	52.0(651)	46.8(167)	
2016	12.7(2222)	12.2(153)	13.2(47)	

[a] Using Pearson chi-square test for categorical variables, ANOVA for continuous variables.

[b] Grouped together due to low N of subjects being self-employed.

Supplementary table 2b. Baseline characteristics by frequency of exposure using propensity score-matched study sample. Presented as % (N) or mean (standard deviation).

Variable	Unexposed (N=1608)	Occasionally exposed (N=1251)	Frequently exposed (N=357)	p-value from tests of difference[a]
**Female**	67.4(1083)	65.3(817)	64.7(231)	0.42
**Age group**				0.71
<u><</u>35	6.9(111)	7.6(95)	7.6(27)	
36-45	21.6(348)	20.7(259)	24.1(86)	
46-55	34.2(550)	33.3(416)	34.2(122)	
56-65	34.6(556)	35.0(438)	32.3(115)	
>65	2.7(43)	3.4(43)	2.0(7)	
**Born in Sweden**	91.0(1463)	89.7(1122)	90.2(322)	0.50
**Married/cohabiting**	72.1(1160)	70.8(886)	70.9(253)	0.71
**Cohabiting with children**	48.0(772)	46.8(586)	48.7(174)	0.75
**Socioeconomic position**				0.99
Unskilled worker	18.8(303)	18.1(226)	19.6(70)	
Skilled worker	16.7(269)	17.3(216)	17.1(61)	
Assistant non-manual employer	13.6(218)	14.0(175)	14.0(50)	
Intermediate non-manual employer	31.2(501)	31.0(388)	30.3(108)	
Professional/upper level executive and self-emplyed[a]	19.7(317)	19.7(246)	19.0(68)	
**Permanent position**	98.6(1586)	98.7(1235)	98.3(351)	0.85
**Job demands (1-5)**	2.9(0.5)	2.8(0.5)	3.0(0.6)	<0.01
**Decision authorithy (1-5)**	2.8(0.8)	2.9(0.8)	2.8(0.8)	0.79
**SA-cmd prior to baseline**	19.5(314)	19.7(246)	23.8(85)	0.17
**Baseline year**				0.41
2012	37.9(609)	35.7(447)	40.1(143)	
2014	50.6(814)	52.0(651)	46.8(167)	
2016	11.5(185)	12.2(153)	13.2(47)	

[a] Using Pearson chi-square test for categorical variables, ANOVA for continuous variables.

[b] Grouped together due to low N of subjects being self-employed.

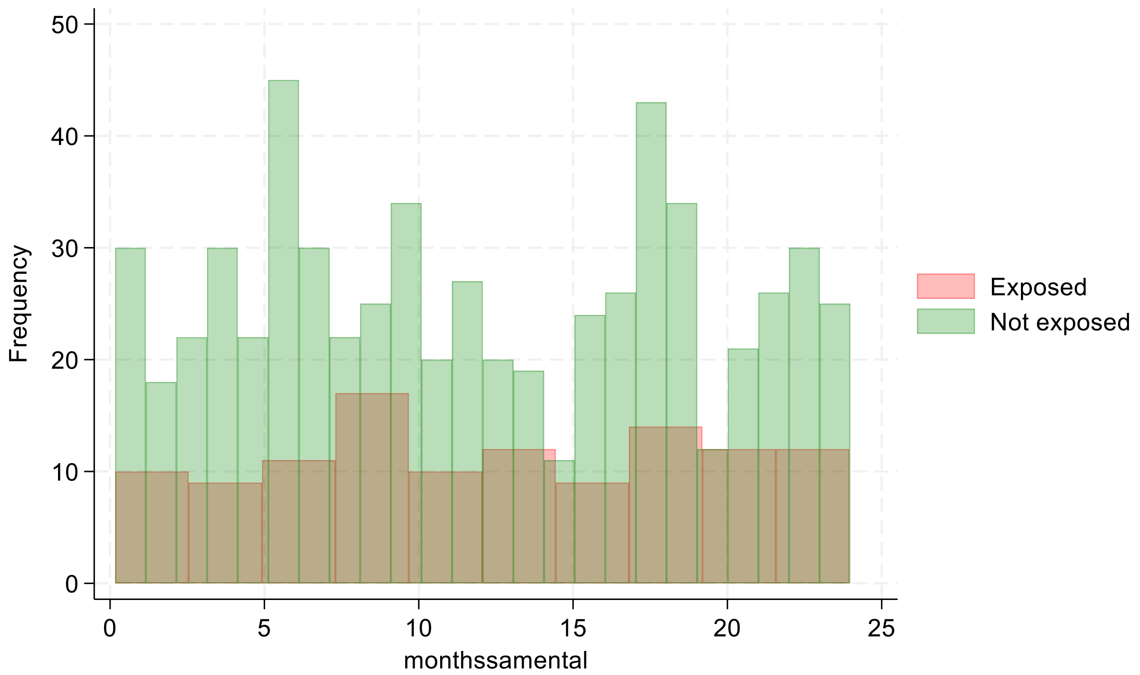

Supplementary figure 4a. Distribution of events of sickness absence due to common mental disorders by time (in months) since baseline survey in full sample.

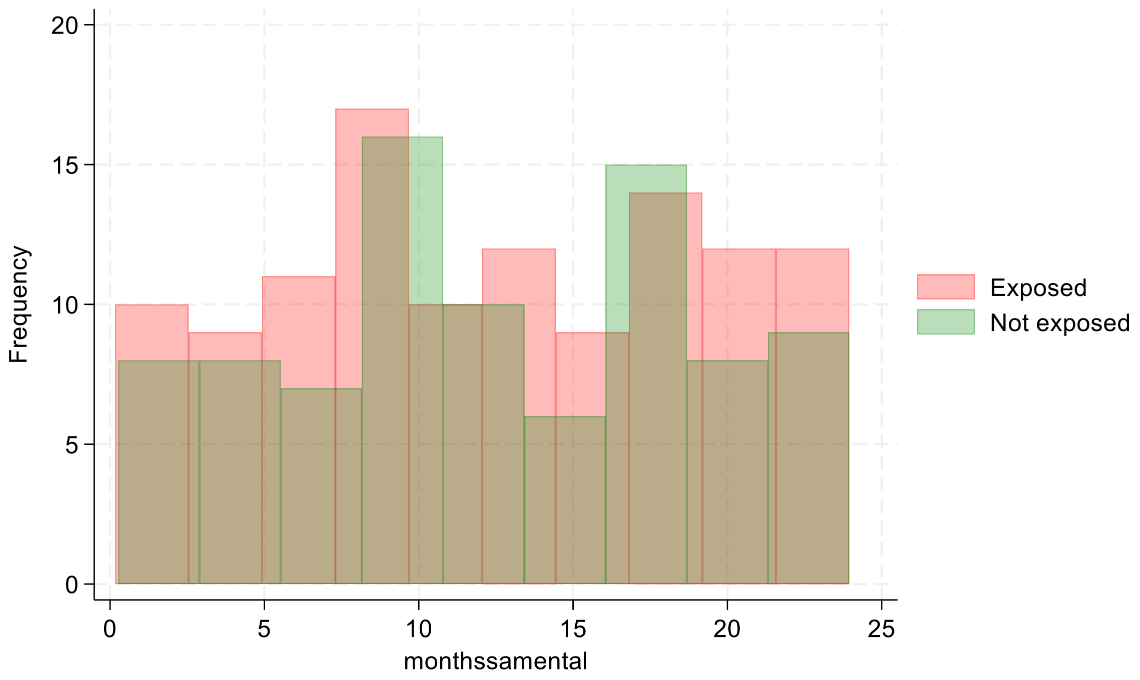

Supplementary figure 4b. Distribution of events of sickness absence due to common mental disorders by time (in months) since baseline survey in propensity-score matched sample.

Supplementary table 3. Hazard rates (HR) with 95% confidence intervals (CI) from Cox proportional hazards models of the association between exposure to workplace bullying and incident sickness absence due to common mental disorders (SA-CMD), using full sample.

	N	Cases SA-cmd	Crude HR (95% CI)	p-value	Adjusted HR[a] (95% CI)	p-value
Occurrence of workplace bullying						
No	17543	616	ref		ref	
Yes	1609	116	2.1 (1.7-2.5)	<0.001	1.4 (1.1-1.7)	<0.01
Frequency of exposure to workplace bullying[b]						
Never	17543	616	ref		ref	
Occasional	1252	83	1.9 (1.5-2.4)	<0.001	1.3 (1.0-1.7)	0.02
Frequent	357	33	2.7 (1.9-3.8)	<0.001	1.6 (1.1-2.3)	0.01

[a] Model adjusted for sex, age, birth country, marital status, cohabiting with children, socioeconomic position, contract type, job demands, decision authority, baseline year and for prior sickness absence due to common mental disorders (SA-cmd, this variable was adjusted for by stratification as to not violate the proportional hazards assumption).

[b] Crude model $p_{trend}<0.001$; adjusted model $p_{trend}<0.01$
